# Computational modeling of light activated ion channels

**DOI:** 10.1186/1471-2202-13-S1-P29

**Published:** 2012-07-16

**Authors:** Roxana A Stefanescu, RG Shivakeshavan, Paul R Carney, Pramod P Khargonekar, Sachin S Talathi

**Affiliations:** 1 Department of Pediatrics, University of Florida, Gainesville, FL 32610, USA; 2 Department of Biomedical Engineering, University of Florida, Gainesville, FL 32610, USA; 3 Department of Electrical Engineering, University of Florida, Gainesville, FL 32610, USA

## 

Channelrhodpsin-2 (ChR2) is a light sensitive ion channel protein currently investigated for millisecond time scale optogenetic control of neural activity [1]. Two competing mathematical models, a 3-state and a 4-state rate transition model are currently available to mimic the ChR2 photocurrent kinetics [2]. While both models are able to capture the key temporal features of ChR2 photocurrent in response to light stimulation pulses of different intensities and durations, little is known about their efficacy to model the neural response to light stimulation protocols of various frequencies and pulse width characteristics. Moreover, it is unclear to what degree the two models can capture the photocurrent kinetics of the recently engineered ChR2 mutants, designed to allow for more precise optical control of neural activity [3].

To address these issues, we investigate a 3-state and a 4-state transition rate model to mimic the photocurrent kinetic of wild type ChR2 (ChR2wt) and a newly validated ChR2 mutant with fast photocurrent kinetics (ChRETA) consistent with the experimental measurements in Gunaydin et al. [3]. We incorporate these models into a fast spiking hippocampal interneuron model [4] in order to examine to what degree the neural response to different experimental stimulation protocols can be successfully simulated.

We find that the 3-state model can qualitatively reproduce the neural activity induced by periodic short time interval (2 ms) light pulse stimulation only for low frequencies (<20Hz). The model however fails to capture the experimentally observed features of neural response to higher frequencies (80 and 200 Hz) for both variants of ChR2 investigated. The 4-state model is able to reliably mimic the neural response to light pulse stimulation of all frequencies in both ChR2 variants. Furthermore, for ChR2wt, the 4-state model but not the 3-state model is able to reproduce both the burst-like neural firing activity induced by single, brief (2ms width) light pulses and the plateau potentials observed in neurons for high frequency (200 Hz) light stimulation. Both models were able to mimic the occurrence of missed spikes in ChR2wt when prolonged (60 light pulses) light stimulation is delivered, with better fidelity provided by the 4-state model. Finally, the 4-state model better captures the neural response to Poisson and Gaussian distributed light stimulation pulses.

Motivated by the overall better performance of the 4-state model, we investigate the conditions under which a characteristic specific to the 3-state model, namely the mono-exponential decay of the ChR2 photocurrent kinetics (often reported in experimental literature) can occur in the 4-state model. Using analytical methods, we show that for each variant, the photocurrent component associated with the unreported decay constant is vanishingly small, which may explain the experimental difficulty in its empirical evaluation.

In summary, we have systematically analyzed the 3-state and 4-state transition rate models for ChR2 photocurrent kinetics and demonstrated that independent of the variant, the 4-state model is able to better capture both qualitative and quantitative features of photocurrent kinetics and the neural response to several different light stimulation protocols. The results suggest that the 4-state transition rate model is a suitable candidate for a universal mathematical framework to model the photocurrent kinetic of ChR2 proteins.
